# A Kindergarten-Based Oral Health Preventive Approach for Hong Kong Preschool Children

**DOI:** 10.3390/healthcare8040545

**Published:** 2020-12-09

**Authors:** Hollis Haotian Chai, Sherry Shiqian Gao, Kitty Jieyi Chen, Duangporn Duangthip, Edward Chin Man Lo, Chun Hung Chu

**Affiliations:** 1 Faculty of Dentistry, The University of Hong Kong, Hong Kong, China; htchai89@hku.hk (H.H.C.); sherryg@hku.hk (S.S.G.); dduang@hku.hk (D.D.); hrdplcm@hku.hk (E.C.M.L.); 2 Hospital of Stomatology, Sun Yat-sen University, Guangdong 510080, China; chenjy679@mail.sysu.edu.cn

**Keywords:** kindergarten, caries, silver diamine fluoride, prevention, children

## Abstract

Early childhood caries (ECC) remains the single most common chronic childhood disease. Untreated caries can cause tooth loss and compromised dentition. Severe ECC can also influence nutrition intake, cognitive development, general health and quality of life. In Hong Kong, approximately half of 5-year-old children suffer from ECC, and more than 90% of these caries remain untreated. Thus, the development of effective strategies for promoting the oral health of preschool children is warranted. The Faculty of Dentistry of the University of Hong Kong has provided kindergarten-based dental outreach services to selected kindergartens since 2008. In 2020, the project expanded to serve all kindergarten children in Hong Kong. The aim of the service is to improve oral health through the prevention and control of ECC among preschool children. The service provides dental screening and silver diamine fluoride treatment for ECC management. In addition, the parents receive oral health talks, and teachers receive training in delivering regular oral health education at kindergarten. The objectives of this service are to improve oral and general health of preschool children, develop the children’s good oral health-related behaviours, maintain the children’s psychological well-being and reduce the burden on their family. This paper describes this kindergarten-based dental outreach service.

## 1. Introduction

**Figure 1:**
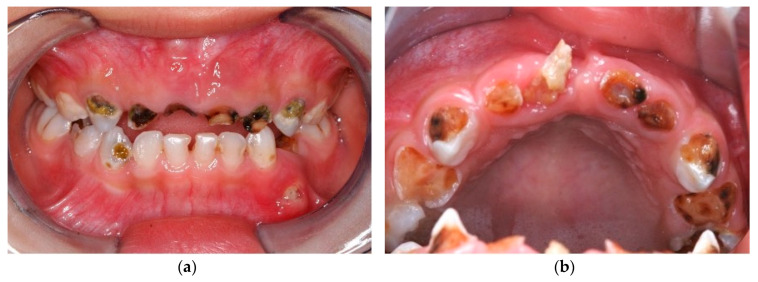
Early childhood caries (ECC) of a 4-year-old Hong Kong child. (**a**) ECC causes pain and infection; (**b**) severe ECC affected this child’s nutrition, growth, physical and psychological development and general health.

Early childhood caries (ECC) is defined as the presence of one or more decayed, missing or filled tooth surfaces in any primary tooth in a child younger than 71 months of age [1]. ECC is the most common chronic childhood disease, and its development has become a global health problem [2]. Because its prevalence has recently increased worldwide in children aged 2 to 5 years, the Fédération Dentaire Internationale (World Dental Federation) has made this age group a priority [3]. ECC causes pain and infection (Figure 1a), and advanced ECC eventually progresses into the tooth pulp to form a dental abscess [4] (Figure 1b). If children’s carious teeth remain untreated, the disease leads to tooth loss and compromised dentition. More importantly, compromised dentition significantly affects the child’s nutrition, as well as their growth, development and general health [5,6]. Infection due to untreated caries can spread systemically. In fact, a child in the United States of America died because the bacteria from a dental abscess spread to his brain [7]. In Hong Kong, a kindergarten-based dental outreach services project aiming at preventing and controlling of ECC among preschool children has been launched to service all kindergarten children in the area. To our knowledge, this is the first community preventive programme using silver diamine fluoride (SDF) reported in the literature. This paper is a detailed communication of this large-scale kindergarten-based dental outreach service project.

## 2. Prevalence of Early Childhood Caries

**Figure 2:**
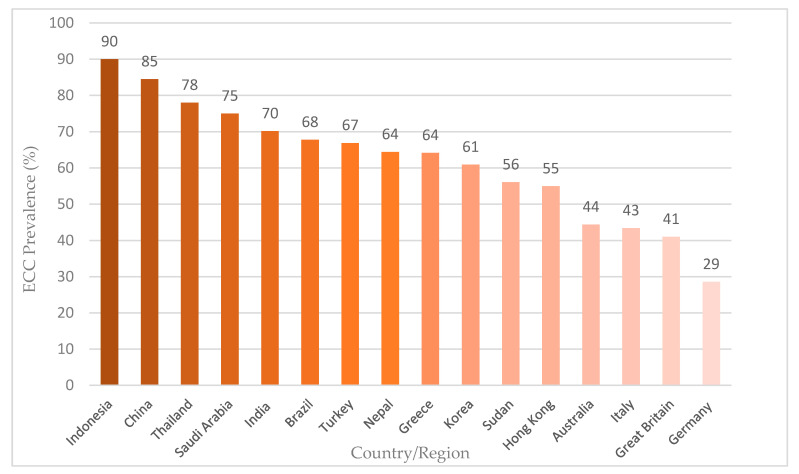
Prevalence of ECC in 5-year-old children (adapted from Chen et al., 2019 [8]).

The World Dental Federation and the World Health Organisation developed the Global Oral Health Goals for the Year 2020 [8]. They have promoted ECC prevention to minimise its impact on young children and their families. ECC can cause a higher risk of new carious lesions. Young children with ECC have higher risk of caries in their permanent dentation compared with those without ECC [4]. Chen et al. [8] performed a systematic review of English publications from 2013 to 2017 to describe the current worldwide prevalence of ECC among 5-year-old children. The review included 37 articles with moderate or good quality surveys conducted in Asia (China, Hong Kong, India, Indonesia, Korea, Nepal and Thailand), Europe (Greece, Germany, Great Britain and Italy), South America (Brazil), the Middle East (Saudi Arabia and Turkey), Oceania (Australia) and Africa (Sudan) (Figure 2).

The majority of the epidemiological surveys found that ECC remained prevalent in young children worldwide [8]. Most publications reported an ECC prevalence higher than 50%, and ECC in young children remains a significant health burden in many countries. The review showed a geographically disproportional distribution of ECC, as the situation in Asian and African countries was unsatisfactory compared to countries in other continents.

**Figure 3:**
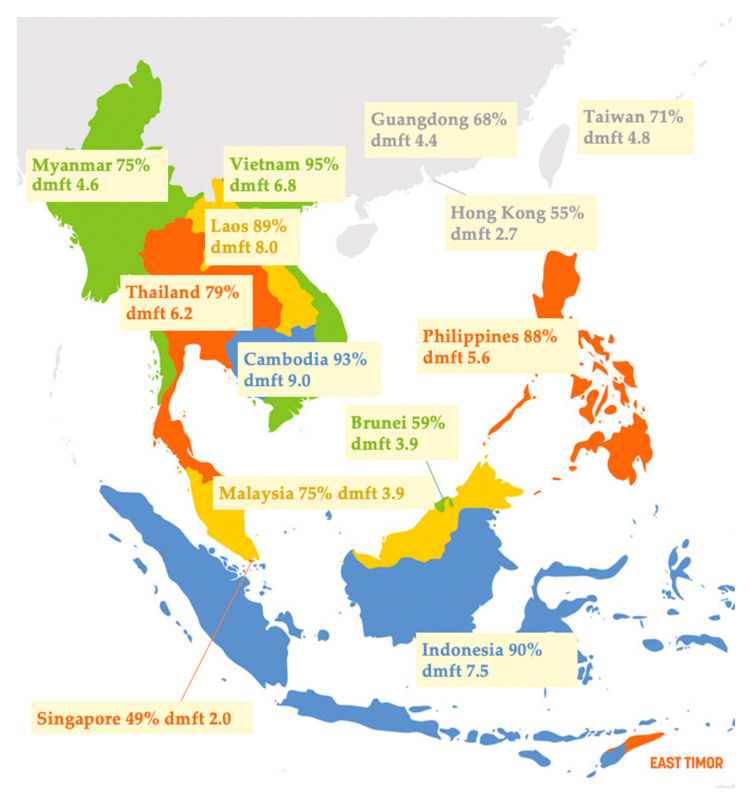
Caries prevalence (%) and caries experience decayed, missing and filled teeth (dmft) of 5-year-old children in Hong Kong and its nearby countries/regions. (adapted from Duangthip et al., 2017; Chen et al., 2019; Li et al., 2020 [8,9,10]).

Another recent review of ECC examined the prevalence and experience of ECC among 5- to 6-year-old children in Southeast Asia [9]. The review retrieved publications from 2006 to 2015 and found that ECC is quite severe and common among Southeast Asian preschool children. Figure 3 summarises the ECC of 5-year-old children in Hong Kong and its nearby countries and regions [8,9,10].

The overall caries status of preschool children in Southeast Asia (median caries prevalence is 79%, whereas caries experience in dmft score is 5.1) is worse than that of such children in other parts of the world. Compared with other countries in Asia, the prevalence of ECC in Southeast Asia is higher than in Hong Kong (51%) and China (66%). Surveys report a much higher prevalence of ECC in Southeast Asia than in developed countries, such as the United Kingdom (28%). Within Southeast Asia, caries prevalence and caries experience in less developed countries (the Philippines, Lao PDR and Cambodia) are higher than those in more developed countries (Singapore and Brunei). In addition, a high number of untreated caries in the primary dentition is almost universal in Southeast Asia. The prevalence and severity of ECC remains substantial in most countries. Thus, more attention should be drawn to reducing the burden of ECC, particularly in less-developed countries.

## 3. Early Childhood Caries in Hong Kong

In recent decades, ECC has remained among the most prevalent non-communicable diseases in children in Hong Kong [11]. Published surveys from the University of Hong Kong and the government’s Department of Health indicated that approximately half of 5-year-old children suffer from ECC and that more than 90% of ECC goes untreated in Hong Kong (Table 1). After monitoring ECC in Hong Kong for more than 20 years, we have concluded that ECC has remained a prevalent local problem for the past two decades.

A 1993 epidemiological survey reported that the prevalence of ECC among 5-year-old children was 63%, with a dmft score of 3.2. The caries prevalence decreased slightly to 51% in a 2001 oral health survey conducted by the Department of Health. However, no significant improvement in the prevalence of ECC has been observed in the past two decades, and the caries status situation remains unsatisfactory in Hong Kong preschool children. Thus, it is necessary to revisit dental public health policies and develop effective evidence-based strategies to promote oral health conditions in Hong Kong preschool children.

Although ECC is severe and prevalent among preschool children in Hong Kong, these children receive no organised dental care. The government’s dental care policy is aimed at raising public awareness of oral hygiene and encouraging proper oral health habits through promotion and education [15]. Through its oral health education unit, the Department of Health provides preschool children with oral health education. Children with ECC can seek dental care from private dentists, but the Department of Health currently does not provide dental treatment to kindergarten children, and the problem of ECC among kindergarten students is becoming serious for 3- (22%) to 5-year-old (55%) children [11,16].

## 4. Social Indicators or Risk Factors of Early Childhood Caries in Hong Kong Children

Periodic surveys that assess community oral health status and oral disease risk factors are essential to subnational oral health surveillance [17]. An epidemiological study was performed to identify common ECC risk factors for 3-year-old children in Hong Kong [16]. The study invited 6331 3-year-old children to participate and examined 5167 of them. The researchers found that both non-modifiable (socioeconomic background) and modifiable (oral health-related knowledge and habits) risk factors were significantly related to ECC. Children, who started brushing their teeth later, had higher snack-intake frequencies, were not born in Hong Kong, had lower monthly family incomes, had lower maternal education levels and had primary caretakers who were not domestic helpers, presented a higher risk of ECC (Table 2).

ECC remains prevalent among preschool children in Hong Kong, and this condition’s occurrence is a complex interaction with various risk factors. Oral health-related behaviours and parental dental knowledge are significantly associated with ECC experience. To address these factors, it is necessary to form partnerships between families and schools. By raising increasing dental knowledge and literacy, enhancing teachers’ ability to deliver oral health education to their students and directly drawing children’s attention to good oral health habits, the promotion of preschool children’s oral health can be achieved. In addition, socioeconomic background is linked with ECC experience. Young children with high ECC risk should be identified early for prevention. However, the majority (75%) of 5-year-old children in Hong Kong have never visited a dentist [14]. Current policies without clinical preventive and curative measures, may not be sufficient to manage the burden of ECC, particularly among those with lower socioeconomic status. The development of an appropriate, cost-effective community prevention service to promote the oral health of preschool children in Hong Kong is warranted. The kindergarten-based dental outreach service provided by the Faculty of Dentistry of the University of Hong Kong is free to all participants.

## 5. Use of Silver Diamine Fluoride for the Management of Early Childhood Caries

ECC management needs a systematic approach. Within primary prevention of ECC, twice daily brushing with fluoride toothpaste is a strategy proven to be effective [18]. Dental professionals can also make efforts to provide instructions of dietary modification and reduction in the use of fermentable carbohydrates among children [19]. In terms of secondary prevention measures, the performance of therapeutic procedures in young children is still a great challenge for dentists, especially when treating child patients who are very young and uncooperative. The lack of a sufficient dental workforce and necessary but sophisticated dental equipment has made it difficult to solve the ECC problem using conventional dental care. An outreach dental care service performed in kindergarten is a pragmatic service for the prevention and control of ECC among kindergarten children. The application of minimally invasive approaches is of utmost importance. Therefore, an alternative strategy has recently been considered for ECC management.

SDF has been proposed for this purpose. It is a clear, odourless ammonia solution containing silver and fluoride ions [20]. The neutral silver fluoride is unstable, so the ammonia acts as a stabilising agent for the solution. Fluoride is effective in enhancing the remineralisation of dental hard tissue, and silver ions act as an antibacterial agent in SDF [21,22]. After the topical application of the SDF solution on a decayed tooth surface, the surface becomes hard and stops progressing. SDF is a safe, effective, efficient, and equitable caries control agent that can be used to help to meet the World Health Organization’s Millennium Goals and to fulfil the US Institute of Medicine’s criteria for 21st-century medical care [21]. A disadvantage of SDF application is the black staining of carious lesions after use. Although many parents have aesthetic concerns about the staining, previous studies found that their acceptance with SDF treatment is still very high upon explanation and that almost all of the treated children and their parents were satisfied with the SDF treatment [23]. To avoid dissatisfaction among participating children and their parents, it is vital to state clearly the black staining after SDF application on informed consent before performing the treatment.

A systematic review with meta-analyses indicated that SDF treatment is non-invasive, simple, quick, painless and effective for managing ECC among young children [24]. The most commonly used concentration of SDF is 38% (44,800 ppm). In addition, the meta-analyses of extracted data from eight SDF clinical trials aimed at arresting caries found that, overall, 81% of treated ECC became hardened (arrested). All eight clinical trials identified as using 38% SDF reported a statistically significant caries-arresting effect on primary teeth. Apart from staining the arrested lesion, no significant complications were reported [24]. The American Academy of Paediatric Dentistry recently set a guideline of using SDF for treating ECC in young children [25]. In addition, the kindergarten-based oral health project that targets Hong Kong preschool children adopted SDF treatment to control ECC.

## 6. A Dental Outreach Service for Hong Kong Kindergarten Children

The Faculty of Dentistry of the University of Hong Kong has been providing kindergarten-based outreach dental service to approximately 20,000 children from 100 kindergartens in a year since 2008. The parental and kindergarten’s acceptance of this service program is very high. Almost 90% of parents agreed to let their children receive SDF treatments if needed. The parents reported no significant complications during these years. The kindergartens were also satisfied with this program, and 97% of them considered this service to be helpful for promoting oral health in young children. These projects have caused a significant decline in the incidence of ECC for participating children from 43% (2010–2011) to 34% (2017–2018).

In 2020, the dental outreach service expanded to serve all kindergarten children in Hong Kong. The aim of the service is to improve oral health through the prevention and control of ECC among kindergarten children. All kindergarten children in Hong Kong are eligible to join this project through their kindergartens. This dental outreach service can serve 1040 kindergartens registered with the Education Bureau, a total of approximately 181,000 children.

The dental outreach service consists of an outreach service aimed at children, oral health promotion for parents and teacher training. The kindergarten-based dental outreach service provides dental screening and SDF treatment to manage ECC. In addition, oral health talks are given to the children’s parents, and teacher training is provided to empower teachers to deliver regular oral health education to kindergarten children at school. The objectives of the service are:

(a) To improve the oral and general health of kindergarten children;
(b) To develop good oral health-related behaviours in children;
(c) To maintain children’s psychological well-being;
(d) To reduce the burden on parents and families.

### 6.1. Dental Outreach Service for Children

**Figure 4:**
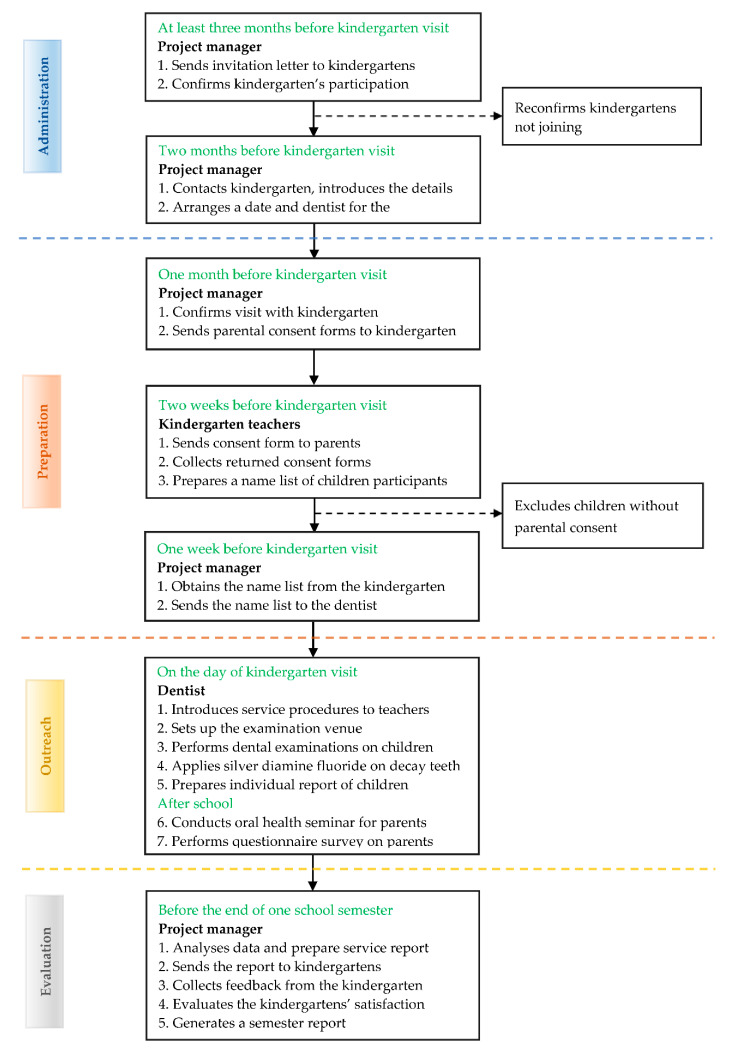
The flow of the kindergarten dental outreach service.

The project’s service flow is shown in Figure 4.

**Figure 5:**
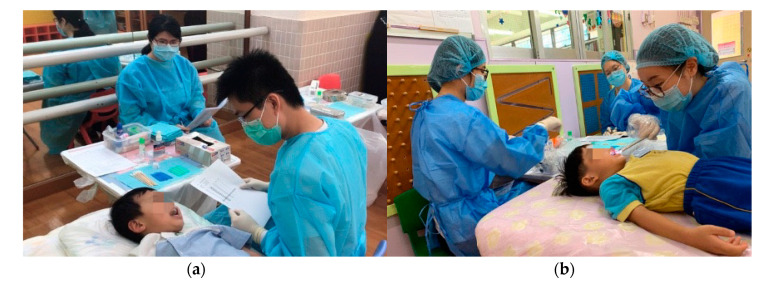
Dental service for children. (**a**) Re-checking a child’s name prior to examination; (**b**) dental examination at a kindergarten.

This project provides a dental outreach service to participating kindergartens on a school day. We send invitation letters and collect written consent from the parents before performing dental examinations and SDF therapy for the children. A reminder is sent to parents before the day of the service. Before providing dental services, we re-check the list of participating children and parental consent with the kindergarten teachers (Figure 5a).

One dentist and one dental surgery assistant (dental nurse) perform the dental service in a classroom or a functional room at the kindergarten. The dentist provides a visual examination for the participating children. If tooth decay is found and the parents accept the SDF treatment, the dentist applies SDF to the decayed tooth. After the examination, we send an individual dental report to the parents regarding their child’s oral health status (Figure 5b).

### 6.2. Oral Health Promotion for Parents

**Figure 6:**
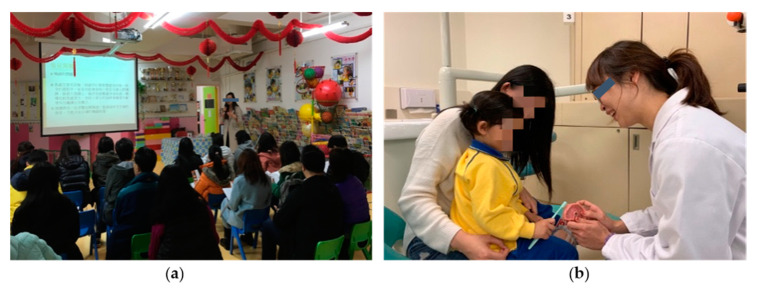
Oral health promotion to parents. (**a**) Oral health education talk for parents; (**b**) individual parental counselling and oral health education.

This project provides oral health seminars to the parents of kindergarten children. The seminars are performed in kindergarten classrooms after school hours, usually on the day of the dental outreach service (Figure 6a). A reminder is sent to the parents. The seminar introduces the dental outreach service, highlights common oral health problems in preschool children, and discusses findings related to the children’s oral health statuses. For example, A question-and-answer session is also held.

The project also provides individual counselling to parents whose children have severe ECC. The content of the individual counselling includes suggestions of dietary modification and instructions of at-home oral healthcare strategies, such as brushing the teeth with fluoride toothpaste. The counselling is conducted in the kindergarten building or at the University of Hong Kong (HKU) Faculty of Dentistry by appointment (Figure 6b). After the counselling, the parents are expected to be able to assist their children in tooth brushing and improve the children’s dietary practices. We encourage parents to visit the Tooth Club website developed by the Oral Health Education Unit, Department of Health.

### 6.3. Teacher Training

**Figure 7:**
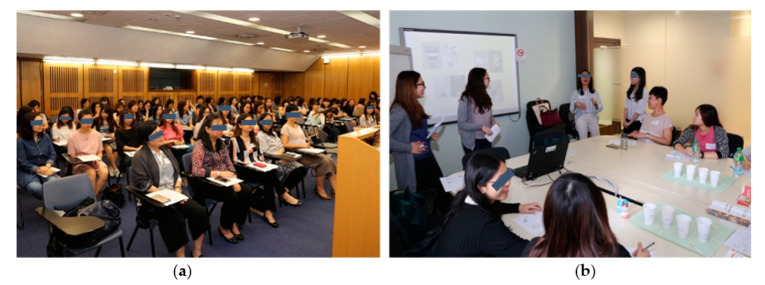
Teacher training. (**a**) Teacher training lecture; (**b**) teacher hands-on workshop.

This project provides teacher training (Figure 7) to all participating kindergarten teachers. The teacher training is aimed at empowering the teachers to deliver effective oral health messages to children in kindergarten.

Lectures, seminars and hands-on workshops are held regularly in the HKU Faculty of Dentistry. Kindergarten teachers gain oral health knowledge and learn about oral healthcare for preschool children. The hands-on workshops have different oral health-related topics, such as brushing the teeth using fluoride toothpaste and dietary instruction. We expect the teachers can adopt these knowledges in their teaching of oral health education in kindergarten. We also provide group discussions on the use of oral health education materials in kindergarten. Participating teachers are divided into small groups for the hands-on workshops and group discussions. We have also developed oral health education materials, including posters, videos and an oral healthcare manual for the kindergarten teachers. Furthermore, we encourage kindergarten teachers to take advantage of online self-learning.

## 7. Project Impact and Merits

### 7.1. Improving Oral and General Health of Kindergarten Children

Considering the significant increase of caries prevalence in 3- to 5-year-old children, the project will protect a large number of teeth from ECC each year. In addition, through SDF treatment, the service will stop ECC from progressing. Unlike untreated ECC, which progresses and becomes unrestorable, arrested or hardened decayed teeth can be restored when the child enters primary school and joins the school dental care service provided by the Department of Health. The service will prevent children from suffering from pain and infection resulting from ECC. Hence, ECC will not adversely affect their growth and development.

### 7.2. Developing Children’s Good Oral Health-Related Behaviours

It is important for children to develop good oral health-related behaviours from a young age. This project plays an important role in encouraging good oral health-related behaviours and attitudes by giving children experience at an early age, educating their parents to provide proper oral health-related practices at home and empowering teachers to provide oral health education in kindergarten. These strategies can raise children’s awareness of oral health. In turn, these strategies will develop good oral health-related behaviours in children.

### 7.3. Maintaining the Psychological Well-Being of Kindergarten Children

ECC prevention will benefit children’s psychological well-being and impart a good quality of life. Children with ECC often present a worse oral health-related quality of life compared to children without ECC. Moreover, sociodemographic variations in oral health-related quality of life are apparent [26]. The project provides the opportunities for those preschool children at high risk of ECC but whose parents cannot afford dental treatment. Thus, by stopping the progress of ECC and helping those children suffering from tooth decay, the oral health-related quality of life of those children and their families can be improved.

### 7.4. Reducing Dental Anxiety and Fear

The project will provide oral health education to children by giving children experience with dental examinations at an early age. Children who have never visited the dentist or who have only visited the dentist in pain are more likely to suffer dental fear [27]. Previous dental experience can affect the development of dental fear and anxiety. The project will introduce awareness to the community members (children, parents and teachers) through dental service and oral health education. Community involvement tends to enhance the instruction of preventive measures and reduce the development of dental fear and anxiety.

### 7.5. Reducing the Burden on the Parents and Family

ECC creates suffering not only for children, but also for their parents and family [28]. ECC can spread systemically, causing fever, and can lead to a substantial number of sick days for children, which also affects their kindergarten education. Moreover, parents or family members must take time to care for the child. Therefore, controlling ECC and keeping the child free from ECC will lower the burden on the child’s parents and family.

## 8. Assessment Plan

This project will invite approximately 181,000 children in all 1040 kindergartens registered with the Education Bureau. We expected at least 800 kindergartens will join this free outreach dental service project. An annual evaluation will be performed. Evaluation will be based on the caries experiences of preschool children and satisfaction of all stakeholders. Data will be collected through charting records, questionnaire and in-depth interviews. The annual evaluation meeting will be held with the participation of the headmistresses of the kindergarten and the representatives of the parent-teacher association. The results of the teachers’ and parents’ satisfaction with the outreach dental service and the findings of the oral health statuses of the children will be discussed. The evaluation results will be summarized and published.

## 9. Remark

More than half of Hong Kong’s 5-year-old children suffer from ECC, and more than 90% of such caries remain untreated. No significant improvement in ECC prevalence has been observed in the past two decades. An epidemiological study also revealed that caries prevalence increases with age during the preschool years. Thus, the identification of high-risk young children and the establishment of cost-effective community prevention services are warranted. A dental outreach project has been providing service to Hong Kong kindergarten children since 2008. In 2020, this dental outreach service expanded to serve all kindergarten children in Hong Kong. The service is aimed at improving oral health through the prevention and ECC control among kindergarten children. The dental outreach service consists of outreach dental services for children (dental screening and SDF treatment), oral health promotion for parents and teacher training. The service’s objectives are to improve the oral and general health of kindergarten children, develop children’s good oral health-related behaviours, maintain children’s psychological well-being and reduce the burden on parents and families. The further evaluation of the project will be based on caries experiences of preschool children and the satisfaction of all stakeholders. We hope this communication can be considered a reference by other dental professionals for planning large-scale community programmes of ECC prevention.

## Figures and Tables

**Table 1:** ECC experience (dmft) and prevalence of 5-year-old Hong Kong children.

Authors, Year [Reference]	Sample Size	Mean dmft	ECC Prevalence
Wei et al., 1993 [12]	1105	3.2	63%
Department of Health, 2001 [13]	3733	2.3	51%
Department of Health, 2011 [14]	1728	2.5	51%
Chen et al., 2017 [11]	505	2.7	55%

dmft: decayed, missing and filled teeth; ECC: early childhood caries.

**Table 2:** ECC in Hong Kong children and associated factors (adapted from Gao et al., 2018 [16]).

Independent Variables	No ECC (dmft = 0)	ECC (dmft > 0)	*p*-Value
Age child began brushing teeth (*n* = 5153)			<0.001
Before 24 months	66% (*n* = 2676)	56% (*n* = 633)	
After 24 months	34% (*n* = 1348)	44% (*n* = 496)	
Daily snack-intake frequency (*n* = 5167)			<0.001
Mean ± SD	2.1 ± 1.2	2.2 ± 1.2	
Birthplace (*n* = 5134)			<0.001
Hong Kong	95% (*n* = 3834)	91% (*n* = 1023)	
Outside Hong Kong	5% (*n* = 180)	9% (*n* = 97)	
Family monthly income (*n* = 5047)			<0.001
Below median income	40% (*n* = 1586)	58% (*n* = 646)	
Above median income	60% (*n* = 2343)	42% (*n* = 472)	
Mother’s education level (*n* = 5096)			<0.001
Mandatory education	21% (*n* = 847)	33% (*n* = 369)	
Higher education	79% (*n* = 3127)	67% (*n* = 753)	
Primary caretaker (*n* = 5172)			<0.001
Parents	65% (*n* = 2625)	76% (*n* = 854)	
Other than parents	35% (*n* = 1420)	24% (*n* = 273)	
